# The Effect of Oligomerization on A Solid-Binding Peptide Binding to Silica-Based Materials

**DOI:** 10.3390/nano10061070

**Published:** 2020-05-30

**Authors:** Rachit Bansal, Zehra Elgundi, Sophia C. Goodchild, Andrew Care, Megan S. Lord, Alison Rodger, Anwar Sunna

**Affiliations:** 1 Department of Molecular Sciences, Macquarie University, Sydney, NSW 2109, Australia; rachit.bansal@hdr.mq.edu.au (R.B.); sophia.goodchild@mq.edu.au (S.C.G.); andrew.care@mq.edu.au (A.C.); alison.rodger@mq.edu.au (A.R.); 2 ARC Centre of Excellence for Nanoscale Biophotonics, Macquarie University, Sydney, NSW 2109, Australia; 3 Graduate School of Biomedical Engineering, University of New South Wales, Sydney, NSW 2052, Australia; z.elgundi@unsw.edu.au (Z.E.); m.lord@unsw.edu.au (M.S.L.); 4 Biomolecular Discovery Research Centre, Macquarie University, Sydney, NSW 2109, Australia

**Keywords:** linker-protein G, oligomerization, solid-binding peptide (SBP), silica-binding peptide, silica-based materials, quartz crystal microbalance with dissipation monitoring (QCM-D)

## Abstract

The bifunctional linker-protein G (LPG) fusion protein comprises a peptide (linker) sequence and a truncated form of Streptococcus strain G148 protein G (protein G). The linker represents a multimeric solid-binding peptide (SBP) comprising 4 × 21-amino acid sequence repeats that display high binding affinity towards silica-based materials. In this study, several truncated derivatives were investigated to determine the effect of the SBP oligomerization on the silica binding function of LPG (for the sake of clarity, LPG will be referred from here on as 4 × LPG). Various biophysical characterization techniques were used to quantify and compare the truncated derivatives against 4 × LPG and protein G without linker (PG). The derivative containing two sequence repeats (2 × LPG) showed minimal binding to silica, while the truncated derivative with only a single sequence (1 × LPG) displayed no binding. The derivative containing three sequence repeats (3 × LPG) was able to bind to silica with a binding affinity of K_D_ = 53.23 ± 4.5 nM, which is 1.5 times lower than that obtained for 4 × LPG under similar experimental conditions. Circular dichroism (CD) spectroscopy and fluorescence spectroscopy studies indicated that the SBP degree of oligomerization has only a small effect on the secondary structure (the linker unravels the beginning of the protein G sequence) and chemical stability of the parent protein G. However, based on quartz crystal microbalance with dissipation monitoring (QCM-D), oligomerization is an important parameter for a strong and stable binding to silica. The replacement of three sequence repeats by a (GGGGS)_12_ glycine-rich spacer indicated that the overall length rather than the SBP oligomerization mediated the effective binding to silica.

## 1. Introduction

Solid-binding peptides (SBPs) are a group of short amino acid sequences that specially bind to the surfaces of various inorganic materials including minerals, semiconductors, and polymers. In doing so, they are able to mediate the simple and controlled attachment of biomolecules onto solid surfaces, conferring biological functionality. SBPs can also act as functionalizing agents for binding and linking biomolecules to solid nanostructures [1,2]. Molecular biology protocols further allow tailoring of the selected peptides to tune their binding and material selectivity properties so that they can be used in bionanotechnological applications. Some SBPs have already been used in the development of multifunctional hybrid materials and for the oriented immobilization of biomolecules [3,4,5].

Proper orientation is usually crucial to preserve the bioactivity, as well as the avidity, of biomolecules. Care et al. demonstrated the oriented immobilization of antibodies onto silica-coated magnetic particles using a bifunctional linker-protein G (LPG) fusion protein, which contains a silica-binding peptide and an antibody-binding region [6]. The binding of SBPs to their corresponding materials is governed by several non-covalent interactions (i.e. electrostatic, polar, hydrophobic, hydrogen bonds, London dispersion) and these interactions typically result in equilibrium binding constants in the nM to sub-mM range [2,7,8]. However, due to the complexity [9,10] displayed at the SBP–material interface [11], many of the mechanisms involved in these interactions remain poorly understood. At present, a collection of experimental and theoretical tools are available to investigate these interactions. For example, surface plasmon resonance (SPR) [12], quartz crystal microbalance with dissipation monitoring (QCM-D) [13], and isothermal titration calorimetry (ITC) [14] can be used to investigate the selectivity, binding kinetics, thermodynamics, stoichiometry, orientation, and viscoelastic properties of these interactions. Circular dichroism (CD) spectroscopy [15], nuclear magnetic resonance (NMR) spectroscopy [16], and Fourier-transform infrared (FTIR) spectroscopy [17] provide information about the structure, conformation and dynamics of SBPs. Computational approaches such as in silico molecular modelling and simulation [18] with the development of artificial intelligence [19,20] provide in-depth knowledge of these interfaces at the atomic level [21].

The interactions between SBPs and their substrates rely on the affinity that particular chemical groups within amino acid residues have for solid surfaces. For example, SBPs that bind to metals predominantly contain hydrophobic and hydroxyl-containing polar residues [7], while those that bind to carbon-based materials are high in aromatic residues [22]. Various molecular-tailoring strategies can be applied to tune the affinity and selectivity of SBPs, the most common being site-specific mutagenesis. Another method used to tune the binding and structural features of an SBP is by increasing the number of repeats of the original SBP sequence, often referred to as multimerization; however, we use the phrase oligomerization to avoid confusion with more commonly used definitions of multimerization. For example, Seker et al. [23] reported the improved affinity and selectivity of a gold-binding peptide (GBP) as a function of polypeptide sequence repeats. Using SPR, they showed that a 3-repeat GBP (3R-GBP) was able to bind five times more strongly to gold than a 1-repeat GBP (1R-GBP). In another case, Cho et al. [24] used ITC analysis to show that a zinc oxide-binding peptide (ZBP) with triplicate tandem repeats (3 × ZBP) displayed a nearly two-fold higher binding affinity (K_D_ = 0.69 µM) to zinc oxide nanoparticles than the single peptide domain (K_D_ = 1.5 µM). These findings implied that there might be a direct relationship between the binding avidity and the number of repeated sequences for some SBPs.

**Figure 1:**
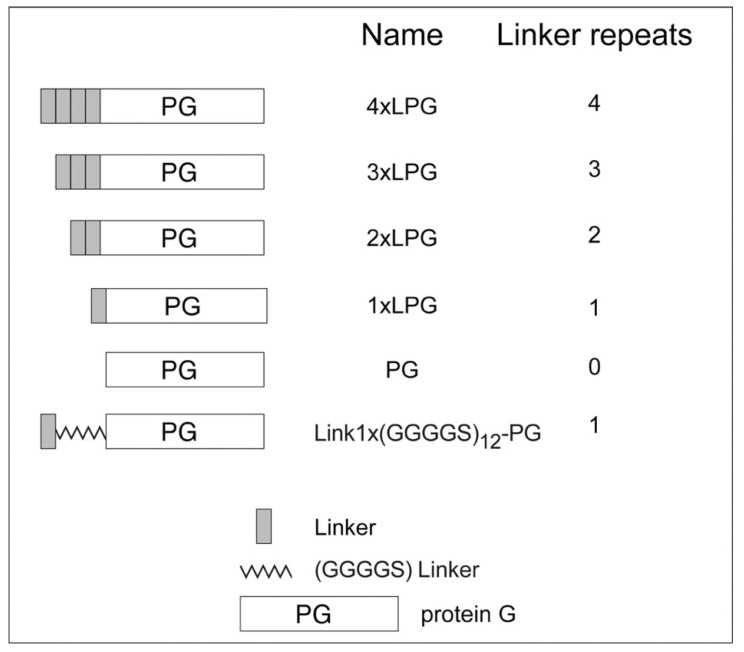
Truncated derivatives used in this study.

LPG (for the sake of clarity, LPG will be referred to from here on as 4 × LPG) was initially reported by Sunna et al. [1] and was designed as a bifunctional fusion protein for the rapid and oriented biofunctionalization of silica-based materials [6,25,26]. 4 × LPG connects a recombinant form of *Streptococcus* protein G antibody-binding protein (PG) with an altered N-terminal domain to a multimeric linker made of a 4 × 21-amino acid sequence repeat (Figure 1) [1] that displays high binding affinity towards silica-based materials. Here, we used various biophysical characterization techniques to examine the silica binding of truncated derivatives of 4 × LPG and to determine the effect of linker oligomerization on the stability of PG.

## 2. Materials and Methods

### 2.1. Materials and Chemicals

The silica binding studies were performed using silica-coated quartz crystals (QSensor QSX 303 SiO_2_) purchased from ATA Scientific (Taren Point, NSW, Australia). Pefabloc, lysozyme, and Benzonase nuclease were purchased from Sigma-Aldrich (Castle Hill, NSW, Australia). Human serum was obtained from Sigma-Aldrich (Castle Hill, NSW, Australia), while the humanized anti-HER2 monoclonal antibody trastuzumab was purchased from Jomar Life Research (Melbourne, VIC, Australia). The human HER2/ErbB2 protein (His-Tag) was purchased from Sino Biological Inc. (Beijing, China). All biological assays were performed at room temperature with standard phosphate-buffered saline (1 × PBS) at pH 7.4 containing 10 mM Na_2_HPO_4_, 1.8 mM KH_2_PO_4_, 137 mM NaCl, and 2.7 mM KCl (Sigma Aldrich, Castle Hill, NSW, Australia). All other chemicals were purchased from Sigma-Aldrich (Castle Hill, NSW, Australia) unless otherwise stated.

### 2.2. Production and Purification of Truncated Derivatives

The expression plasmids (pLink1 × pET22b, pLink2 × pET22b, and pLink3 × pET22b) used to produce the recombinant proteins were previously reported by [1]. The production of each of the truncated derivatives (1 × LPG, 2 × LPG, and 3 × LPG) was as follows: 1 L Luria Bertani (LB) medium (Merck Millipore, Bayswater, VIC, Australia) supplemented with 50 µg/mL carbenicillin was inoculated with 10 mL of an overnight culture of *E. coli* Tuner (DE3) cells (Novagen) harbouring the expression plasmid. The culture was incubated at 37 °C with continuous shaking (250 rpm) until the A_600_ was approximately 0.7–1.0. The incubation temperature was reduced to 20 °C and protein synthesis was induced by the addition of 0.2 mM isopropyl β-D-thiogalactoside (IPTG). Cells were harvested after 3–4 h induction by centrifugation for 15 min at 10,000× *g* and 4 °C and were stored at −20 °C.

The cells were resuspended in ice-cold lysis buffer (25 mM Tris–HCl, pH 8.0, 100 mM NaCl, 1.25 mM EDTA, and 0.05% Tween 20), supplemented with 4 mM of the Pefabloc serine protease inhibitor and 1.5 mg lysozyme. They were ruptured by three passages through a French pressure cell (Thermo Fisher Scientific Australia, Scoresby, VIC, Australia). After cell rupture, 50 units of Benzonase nuclease and 4 mM Pefabloc were added. The sample was incubated on ice for 20 min. The debris was removed by centrifugation for 30 min at 20,000× *g* and 4 °C. The supernatant obtained was first filtered through a 0.45 µm and then 0.22 µm sterile filter and stored at 4 °C.

All purifications were performed using the Äkta™ start chromatography system (GE Healthcare, Parramatta, NSW, Australia). For purification, each truncated derivative soluble extract (1 × LPG, 2 × LPG, and 3 × LPG) was loaded onto a 5 mL HiTrap Q anion exchanger column (GE Healthcare) previously equilibrated with 25 mM Tris–HCl, pH 8.0, supplemented with 100 mM NaCl. The column was washed extensively with the same buffer. Under these conditions, all truncated derivatives eluted at 200 mM NaCl. The eluted protein samples were concentrated using an Amicon Ultra-15 centrifugal filter (10 kDa cut-off, Merck Millipore) followed by buffer exchange with a PD-10 desalting column (GE Healthcare). In addition, 1 × LPG was buffer exchanged into 20 mM Bis-Tris (bis-(2-hydroxyethyl)-amino-tris(hydroxymethyl)-methane) buffer pH 6.0. This fraction was applied again onto the 5 mL HiTrap Q column (GE Healthcare) previously equilibrated with 20 mM Bis-Tris buffer pH 6.0. The column was washed extensively with the same buffer and the 1 × LPG recombinant protein was eluted at 200 mM NaCl. 2 × LPG and 3 × LPG were buffer exchanged into 50 mM HEPES (4-(2-hydroxyethyl)-1-piperazineethanesulfonic acid) buffer pH 8.0. The fractions were applied to a 5 mL HiTrap SP cation exchanger column previously equilibrated with 50 mM HEPES buffer pH 8.0. The column was washed extensively with the same buffer. Under these conditions, 2 × LPG eluted at 50 mM NaCl, while 3 × LPG eluted at 100 mM NaCl.

Fractions containing the truncated derivatives were identified on 4–15% Mini-PROTEAN^®^ TGX™ Precast Protein Gels (Bio-Rad Laboratories, Gladesville, NSW, Australia) by sodium dodecyl sulphate (SDS)-polyacrylamide gel electrophoresis (PAGE) and stained with Coomassie Brilliant Blue. Individual fractions were concentrated using an Amicon Ultra-15 centrifugal filter and all final samples were stored in 1 × PBS buffer (after fresh addition of Pefabloc) at –80 °C.

The final protein concentration was measured using the Micro BCA protein assay kit (Thermo Fisher Scientific Australia, Scoresby, VIC, Australia) according to the manufacturer’s instructions.

### 2.3. Construction, Production, and Purification of a Synthetic Linker Derivative

Three sequence repeats from the 4 × LPG peptide linker were replaced by a synthetic (GGGGS)_n_ linker sequence. This replacement was important to determine whether the oligomer or simply the distance from the PG was required for efficient binding to silica. The PG sequence was ligated into plasmid pET44a (+) via NheI/BamHI restriction sites to obtain the plasmid pET44-PG. A gene sequence containing one original SBP sequence from LPG followed by the synthetic linker sequence (GGGGS)_12_ was synthesized by Invitrogen GeneArt Gene Synthesis (ThermosFisher Scientific Australia) and ligated into plasmid pET44-PG via NdeI/NheI restriction sites to assemble the expression plasmid pLink1 × (GGGGS)_12_-PG. *E. coli α*-Select (Bioline, Eveleigh, NSW, Australia) was used as a host for general gene cloning and vector storage, and *E. coli* Tuner (DE3) cells were used for recombinant protein expression.

Link1 × (GGGGS)_12_-PG was produced and the soluble protein fraction was obtained as described for the truncated derivatives. For purification of Link1 × (GGGGS)_12_-PG, the soluble extract was loaded onto a 5 mL HiTrap Q anion exchanger column previously equilibrated with 25 mM Tris–HCl, pH 8.0, supplemented with 100 mM NaCl. The column was washed extensively with the same buffer. Under these conditions, the Link1 × (GGGGS)_12_-PG did not bind to the column and was found in the unbound fraction. The Link1 × (GGGGS)_12_-PG was concentrated using an Amicon Ultra-15 centrifugal filter (10 kDa cut-off) followed by buffer exchange with a PD-10 desalting column and 50 mM MES (2-(N-morpholino) ethanesulfonic acid) buffer pH 6.0. This fraction was applied to a 5 mL HiTrap SP cation exchanger column previously equilibrated with 50 mM MES buffer pH 6.0. The column was washed extensively with the same buffer and the Link1 × (GGGGS)_12_-PG was eluted at 300 mM NaCl. Fractions containing the Link1 × (GGGGS)_12_-PG were identified by SDS-PAGE as described above. Fractions containing the purified Link1 × (GGGGS)_12_-PG were pooled and concentrated using an Amicon Ultra-15 centrifugal filter. Samples were stored in 50 mM MES buffer pH 6.0 at –20 °C after sterile filtration through a 0.22 µm filter.

### 2.4. Silica Binding Assay

The binding of 4 × LPG and the truncated derivatives used in this study (3 × LPG, 2 × LPG, 1 × LPG, and PG) to silica was previously reported by [1] using a silica binding assay. Similarly, the binding of purified recombinant Link1 × (GGGGS)_12_-PG to silica was determined as follows: 5 mg silica (BDH Ltd., Poole, Dorset, U.K.) was washed three times with washing buffer (10 mM Tris–HCl, pH 7.5, 100 mM NaCl, and 1% Triton-X100). Soluble protein in a final volume of 100 µL was mixed with silica and incubated by rotation at room temperature for 1 h. The unbound fraction was removed after centrifugation at 14,000× *g* for 20 s. The silica pellet was washed three times by vortexing with 100 µL of 100 mM Tris–HCl buffer, pH 8.0. Finally, the silica-bound protein was eluted after addition of 100 µL of SDS PAGE-loading buffer and incubation at 99 °C for 10 min (with short mixing every 2 min). Fractions containing the Link1 × (GGGGS)_12_-PG protein were identified by SDS-PAGE as described above.

The percentage of protein in the different fractions was semi-quantified by analysing the intensity of bands of digital images from SDS-PAGE with ImageJ software (version 1.48, NIH, Bethesda, MD, USA) (http://rsb.info.nih.gov/ij/index.html).

### 2.5. Circular Dichroism (CD) Spectroscopy

CD spectra were collected at room temperature (approximately 25 °C) on a JASCO J-1500 spectrophotometer (JASCO Corporation, Japan). CD data were collected in water in order to avoid any interference or noise below 200 nm as PBS absorbs strongly at wavelengths below 200 nm. All protein samples were buffer exchanged to Milli-Q water using Amicon Ultra-10K 0.5 mL centrifugal filters and were then further diluted in Milli-Q water to a final concentration of 0.1 mg/mL (as determined by the Micro BCA protein assay). Wavelength scans were performed between 180 and 350 nm in a rectangular, 1 mm pathlength quartz cuvette (Starna Scientific Ltd., Ilford, UK). For each sample, 3 accumulations were recorded using a 2 nm bandwidth, a scan speed of 100 nm/min, and a digital integration time (D.I.T.) of 2 s. The data are reported in terms of molar extinction coefficient (Δɛ), expressed in mol^−1^ cm^−1^ dm^3^.

### 2.6. Circular Dichroism Secondary Structure Analysis

Circular dichroism spectra were analysed using a graphical user interface (GUI) version (GUI to Implement SOMSpec, a CD Secondary Structure Fitting Approach 2018, gitlab.com of our validated self-organizing map approach to secondary structure fitting [27,28]. In this approach, a reference set, in our case, SP175 [29] (truncated to 190 nm), augmented with a constructed 100% helix and 100% random coil peptide data [30] is organized into a map with spectra of similar shape in the same neighbourhood. Another map, which overlays the first, contains the secondary structure contents of the reference proteins and the nodes whose secondary structure is determined by interpolation from the neighbouring 5 references spectra. An unknown spectrum is placed on the map where it minimizes the distance (difference) between it and the spectral shape and magnitude of a nodal spectrum. The unknown protein’s secondary structure is then assigned to be that of the node. We ran SOMSpec with a 50 × 50 map, 5 best matching units, and a wavelength range from 240 to 190 nm. The goodness of fit of the secondary structure estimates was assessed by considering the overlay of the experimental and predicted spectrum visually and by a normalized root-mean-square deviation (NRMSD). Visual inspection weights the maxima, minima, and where a spectrum crosses the zero-line more than other points in the spectrum, whereas the NRMSD approach equally weights all wavelengths. In general, for spectra from 240 to 190 nm, an NRMSD < 0.02 indicates a good fit with only 1–2% uncertainty, whereas higher NRMSDs indicate more uncertainty in the prediction. Higher values usually require user interference to choose the best fit.

It was visually apparent (from the shift in the negative maximum from 208 nm towards 200 nm) in the spectra that addition of longer peptides increased the unfolded percentages of the proteins. However, the fitting method was not selecting the random coil member of the reference set, due to it not being one of its best matching units. We, therefore, manually subtracted different fractions of the random coil spectrum for the SufI-KK peptide MSLSKKQFIQASGIALCAGAVPLKASA [30] and reran SOMSpec to obtain the best possible fit. The derandomized molar residue Δ*ε* was determined from the experimental Δ*ε* using

(1) $$\Delta \varepsilon_{\mathrm{derandomised}}=\frac{\Delta \varepsilon_{\exp}-\frac{{\#}_{RC}}{{\#}_{\exp}}\Delta \varepsilon_{RC}}{1-\frac{{\#}_{RC}}{{\#}_{\exp}}}$$

where *RC* denotes random coil, # denotes the number of residues, exp denotes the full protein on which data were collected, and derandomized denotes the core of the protein when the linkers and any extra unfolded residues are removed. The derivation of Equation (1) is provided in Equation (S1) (Supplementary Materials).

### 2.7. Fluorescence Spectroscopy

Protein samples were dissolved to a final concentration of 2 µM in 1 × PBS containing various concentrations (0–7 M) of guanidinium hydrochloride (GdnHCl). All measurements were recorded at room temperature (approximately 25 °C) in a micro-volume fluorescence cuvette with a 3 mm pathlength (Starna Scientific Ltd., Ilford, UK) using a JASCO FP-8500 Spectrofluorometer (JASCO Corporation, Japan). Fluorescence emission spectra were collected between 300 and 550 nm using a 295 nm excitation wavelength, 2.5 nm excitation bandwidth, 5 nm emission bandwidth, and scan speed of 100 nm/min.

### 2.8. Quartz Crystal Microbalance with Dissipation Monitoring (QCM-D) Interaction Analyses

The adsorption of the proteins (1 × LPG, 2 × LPG, and 3 × LPG) onto silica was monitored on a 4-channel QSense Analyzer QCM-D (Biolin Scientific AB, Gothenburg, Sweden) using SiO_2_-coated sensors. Sensors were cleaned before use with 2% sodium dodecyl sulphate (SDS) for 30 min at room temperature, washed thoroughly with Milli-Q water, and dried with nitrogen gas prior to use. The temperature of the QCM-D was maintained at 22 ± 0.1 °C and the flow rate was set to 150 µL/min throughout all experiments. Sensor crystals were exposed to 1 × PBS, and stable frequency (*f*) and dissipation (D) measurements were established. Test protein solutions were prepared in 1 × PBS buffer pH 7.4 at room temperature at concentrations of 3.27–654 nM and exposed to the surfaces for 900–15,000 s and then rinsed with 1 × PBS until consistent *f* and D measurements were obtained. In addition, the ability of an antibody and antigen complex to bind to 3 × LPG immobilized on silica was explored. Sensor crystals were exposed to 1 × PBS, and stable *f* and D measurements were established. 3 × LPG was exposed to silica at a concentration of 3.27–654 nM for 900–15,000 s, and the sensors were then rinsed with 1 × PBS until consistent *f* and D measurements were obtained. To eliminate the risk of non-specific binding, the free binding sites on the crystal surface were blocked by injecting 1 mg/mL BSA (in 1 × PBS) followed by rinses by rinsing with 1 × PBS until stable *f* and D measurements were obtained. The humanized anti-HER2 monoclonal antibody, trastuzumab, was then injected into the system at a final concentration of 1 µg/mL until a stable response was observed followed by rinses by rinsing with 1 × PBS until stable *f* and D measurements were obtained. The final step included the injection of HER2 antigen at a concentration of 1 µg/mL until equilibrium was reached.

A minimum of three adsorption curves were recorded for each test protein at each concentration, which were measured at the fundamental *f* (5 MHz), as well as the third (15 MHz) to eleventh (55 MHz) overtones. All solutions were passed through a 0.22 μm filter and degassed at room temperature before use. The adsorbed mass is proportional to the change in *f* (∆*f*) via the Sauerbrey equation only when the adsorbed layer is rigid, uniformly distributed on the sensor surface, and the adsorbed mass is small compared to the mass of the crystal [31,32]. However, in the case of most proteins, these assumptions are not valid, as the adsorbed layer is viscoelastic as there is a dissipation change greater than 1 × 10^6^/10 Hz. Under these conditions, the Voigt viscoelastic model is used to estimate the adsorbed protein layer mass and thickness from *f* and D measurements at multiple harmonics, assuming an adsorbed protein layer density of 1000 g/m^3^ [33,34,35]. The Voigt model was applied in the QTools software (QSense). Data are presented as ∆*f* and ∆D versus time graphs or D*f* plots (∆D versus ∆*f*) for the third overtone.

## 3. Results and Discussion

### 3.1. Effect of Linker Oligomerization on the Secondary Structure of PG

**Figure 2:**
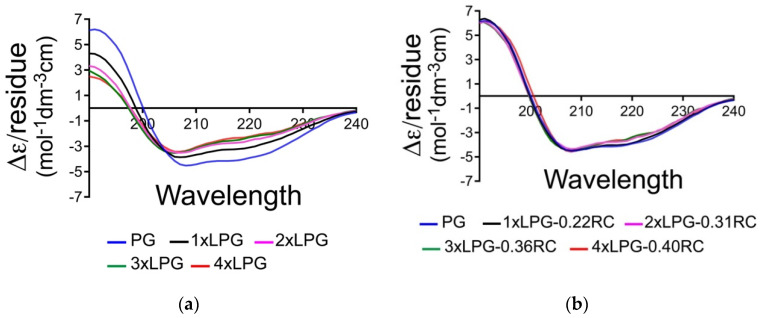
(**a**) Far-UV Δ*ɛ*/residue spectra of protein G (PG) (blue), 1 × linker-protein G (LPG) (black), 2 × LPG (pink), 3 × LPG (green), and 4 × LPG (red) in water collected at a concentration of 0.1 mg/mLl. (**b**) De-randomized Δ*ɛ*/residue spectra with the indicated percentage of a peptide random coil spectrum subtracted according to Equation (1).

The bioactivity of a biomolecule is dependent on its secondary structure. CD spectroscopy was used to determine the effect of different truncations of the linker sequence on the overall secondary structure of PG. A concentration of 0.1 mg/mL (the absorbance for the CD signals measured between 180 and 350 nm was normalized to account for the small variations in concentration) was used to measure the far-UV spectra of the truncated derivatives (1 × LPG, 2 × LPG, and 3 × LPG). As shown in Figure 2a, the presence of a positive peak at ~190 nm and negative peaks at ~208 and ~222 nm indicates significant α-helical content in all the proteins, which is evident. The SOMSpec secondary structure estimates using an augmented SP175 reference set (truncated to be from 240 to 190 nm) gave the secondary structure estimates of the first section of Table 1. The NRMSDs and the SOMSpec output (illustrated in Figure S1, Supplementary Materials) indicate that the PG analysis is of satisfactory quality with 38% α-helix and 16% β-sheet. Goward et al.’s [36] prediction of 37% + 4% α-helix and 30% ± 5% β-sheet supports this helix estimate and raises doubts about the sheet. Their CD analysis (using CONTIN) [37] suggested approximately 10% less helix and 10% more sheet. CONTIN uses a ridge regression procedure, which fits the CD spectrum of the test protein as a linear combination of the CD spectra of N reference proteins by minimizing a function. CONTIN was deemed by Sreerama and Woody to seldom give the best fit [38]. The 2IGG Protein Data Bank (PDB) NMR structure [39] suggests that the 64-residue IGG-binding domain that is repeated in PG is only 23% α-helix but 37% β-sheet. However, the N-terminal β-strands are curved into a 2D helix in the PDB structures, would need to be unpacked to form PG, and have high propensity helix residues on the N-terminal side of the helix. Thus, we believe our secondary structure estimate for PG is reasonably accurate. By way of contrast, both the NRMSD and visual inspection of the SOMSpec results for 1 × LPG, 2 × LPG, 3 × LPG, and 4 × LPG are far from satisfactory (Table 1 and Figure S1, Supplementary Materials).

We, therefore, subtracted the CD of different fractions of a random coil polypeptide, selected the fraction that had the best SOMSpec predicted spectrum, and determined the secondary structure contents. Figure 2b shows the overlay of the derandomized spectra. While not perfect, reflecting the somewhat arbitrary choice of a peptide random coil spectrum, it is sufficient for our purposes. Table 1 part 2 shows the SOMSpec output for the best fits of the derandomized proteins (fraction of random coil removed is indicated). Part 3 indicates the secondary structure content when the full random coil content is included. For each of the linker proteins, the amount of random coil required to obtain the best fit is about 14% of the residues of PG plus the appropriate linker. Although there is an error to the fitting, our data suggest that the addition of the linkers (and removal/replacement of some of the N-terminal PG residues) not only adds unstructured protein but also causes the core protein to lose some structure: ~10 residues of helix, ~6 of sheet for 1 × LPG, and more for the longer ones (Table 2). This supports the C’ domain structure prediction of Goward et al. [36] with its helix near the N-terminal (and thus supports our PG structure estimates above). The loss of structured residues increases slightly for the longer linkers (Table 2).

### 3.2. Effect of Linker Oligomerization Chemical Stability of PG

**Figure 3:**
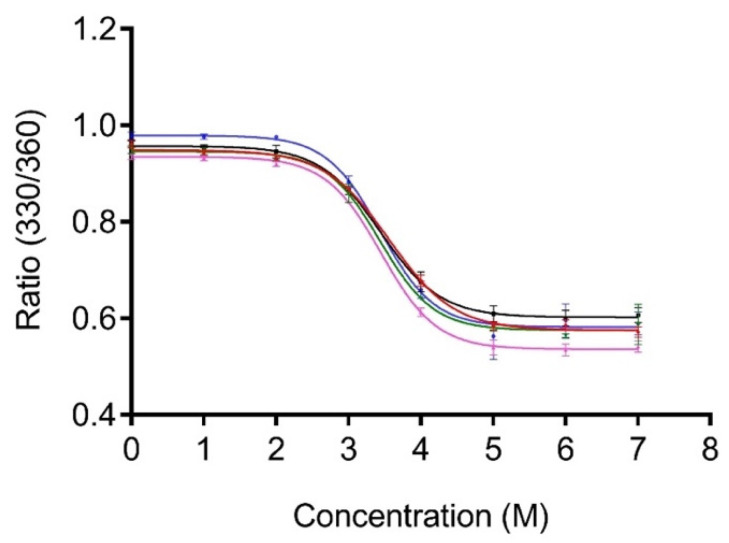
Unfolding of PG (blue), 1 × LPG (black), 2 × LPG (pink), 3 × LPG (green), and 4 × LPG (red) in the presence of guanidinium hydrochloride (GdnHCl). All protein samples were diluted to a final concentration of 2 µM in 1 × PBS, pH 7.4, containing various molar concentrations of GdnHCl. The ratio of relative fluorescence intensity at 330 and 360 nm (330/360) was monitored after excitation at 295 nm.

The relative stability of all truncated derivatives was compared in the presence of the chemical denaturant GdnHCl using fluorescence spectroscopy. PG includes eight tyrosine and three tryptophan residues in the core of the protein. Accordingly, intrinsic tryptophan fluorescence (ITF) experiments were performed at an excitation of 295 nm to eliminate the contribution of tyrosine to the measured fluorescence. The linker sequence does not contain any aromatic residues, so it does not contribute to the overall fluorescence. The unfolding of 1 × LPG, 2 × LPG, 3 × LPG, and 4 × LPG was monitored by measuring the shift in the maximum tryptophan fluorescence emission with an increasing concentration of GdnHCl, and the ratio of the fluorescence intensities at 330 and 360 nm was plotted against increasing denaturant concentration (Figure 3).

Assuming the protein denaturation to be a two-state process, GraphPad Prism 7.0 (GraphPad Software, La Jolla, CA, USA) was used to non-linearly fit the data. The concentration of GdnHCl required for 50% unfolding of the core protein where the tryptophans are located was determined to be 3.4 ± 0.2 M (95% CI), 3.4 ± 0.1 (95% CI), and 3.4 ± 0.2 (95% CI) for 1 × LPG, 2 × LPG, and 3 × LPG, respectively. The concentrations for PG and 4 × LPG were previously reported as 3.4 ± 0.2 M and 3.6 ± 0.1 M, respectively [31]. Thus, the chemical stability of the core PG portion of the fusion proteins is, within experimental error, unaffected by the linker sequences.

### 3.3. Minimal Number of Repeats Required for Silica Binding

**Figure 4:**
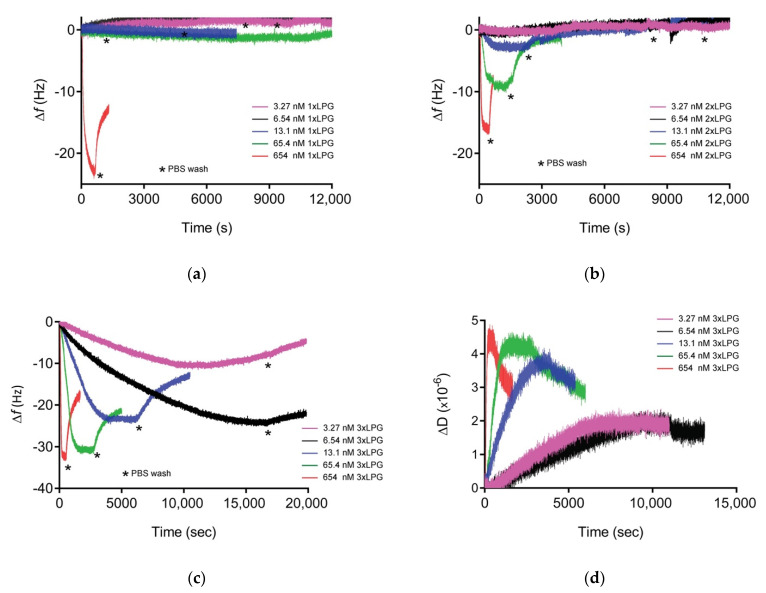
Representative Δ*f* vs. time sensorgrams for (**a**) 1 × LPG, (**b**) 2 × LPG, and (**c**) 3 × LPG binding to silica, and ΔD vs. time for (**d**) 3 × LPG binding to silica as measured at the 3^rd^ overtone by quartz crystal microbalance with dissipation monitoring (QCM-D) crystals at various concentrations (3.27–654 nM). The measurements were performed at a flow rate of 150 µL/mL at 22 °C in three steps: Establishment of baseline (1 × PBS buffer), adsorption of protein until saturation, and washing (1 × PBS) to remove unbound protein. Three independent measurements were performed for each concentration.

The binding affinity of the various truncated derivatives to a silica surface was determined using QCM-D (Figure 4). The binding affinity of 1 × LPG was beneath the limit of quantitation as only the highest concentration showed some binding, but the adsorbed protein was removed after washing with 1 × PBS (Figure 4a ∆*f* vs. time sensorgram, Supplementary Figure S2a, Supplementary Materials). The sensorgram for 2 × LPG and a silica surface (Figure 4b) showed measurable binding at the three highest protein concentrations (654, 65.4, and 13.1 nM). Although it was not possible to calculate the binding affinity for 2 × LPG, the frequency shift for the highest 2 × LPG concentration suggests that the adsorption of the protein to the silica surface is almost half (~16.5 Hz) that previously reported observed for 4 × LPG [31]. Binding of 3 × LPG to silica was observed at all concentrations analysed with both changes in *f* and D measurements observed (Figure 4c,d) and a binding affinity (K_D_) of 53 ± 5 nM, when fitted using the Langmuir isotherm (Figure S3, Supplementary Materials). This K_D_ value is 1.5 times higher than that obtained for 4 × LPG under similar experimental conditions (K_D_ = 35 ± 12 nM), indicating an overall lower binding affinity of the 3 × LPG truncated derivative. Based on these data, a general trend of increased binding to the silica surface was observed with linker oligomerization.

The D*f* plot generated from the QCM-D data for 3 × LPG binding to silica (Figure S4, Supplementary Materials) indicated that the data were contained within the viscoelastic region consistent with the need to utilize the Voigt viscoelastic model to estimate bound thickness and mass and estimate the viscosity of the adsorbed layer (Table 3). The viscous nature of the adsorbed protein layer might be due to the entrapment of water between the peptide layers owing to the hydrophilic nature of the protein. This was also the case for the transient binding between either 1 × LPG or 2 × LPG and silica with ∆D values greater than 1 × 10^6^/10 Hz (Supplementary Figure S2, Supplementary Materials), although the binding was not strong to withstand rinsing with 1 × PBS with 1 × LPG completely removed from the surface after the PBS washing step, and only half of the initially bound 2 × LPG remained on the surface after the washing step.

The maximum thickness of the peptide layer was calculated by the Voigt model at the point of maximum ∆*f* prior to rinsing with 1 × PBS and was found to be 6 nm for 3 × LPG binding to silica, which equated to a layer with a mass of approximately 680 ng/cm^2^ (Table 3). Analysis of the orientation of 3 × LPG on the silica surface was determined by taking into account its molecular weight of 28.2 kDa and globular nature, as performed previously for 4 × LPG [34], and was approximated to be 324 and 175 ng/cm^2^ for end-on and side-on orientation, respectively. The mass of 3 × LPG on the silica surface was approximately 680 ng/cm^2^ (Table 3), which, based on the analysis above, is most likely a hydrated end-on configuration, as observed previously for 4 × LPG [31]. Based on these results, it appears that the three repeats of the linker (3 × LPG) support a similar level of binding as 4 × LPG, but with comparatively weaker binding affinity.

Initial work by Brown highlighted the possible dependence of a gold-binding peptide binding and avidity towards its inorganic substrate to the peptide oligomerization [40]. In this case, the binding affinity of a gold-binding peptide and alkaline phosphatase fusion protein to gold was directly dependent on the numbers of repeating gold-binding polypeptides. Seker et al. [32] studied the effect of SBP oligomerization on the binding affinity to their solid substrate. An increase in the binding affinity of a gold-binding peptide (GBP1) was observed with the increasing number of tandem repeats (3-repeats). Similarly, increased binding affinity to the surface of ZnO-coated nanoparticles was observed after the oligomerization of a zinc-binding peptide (ZBP). In this case, the trimeric (3 × ZBP) variant displayed a binding to the ZnO-coated nanoparticles nearly twice as strong as that observed with the monomeric (1 × ZBP) variant [24]. Interestingly, the binding stoichiometry of each ZBP per ZnO-coated nanoparticle followed a reverse pattern where it was lower for 3 × ZBP (~2.3 molecules) as compared to 1 × ZBP (~3.9 molecules). This can be attributed to the fact that the small tethered length of 1×ZBP supported a denser packing of the peptide molecules onto the ZnO surface due to the availability of comparatively more free binding sites. The silica-binding peptides QBP1 and QBP2 displayed equilibrium binding constants of 0.12 × 10^6^ and 1.2 × 10^6^, respectively [32]. However, only the tandem 3-repeats derivative of QBP1 (3l-QBP1) showed increased affinity to silica but not the QBP2 derivative (3l-QBP2).

Based on the current data available, there is no general trend for increased binding strength as a result of SBP oligomerization. While in some cases, oligomerization increased the binding affinity, in others, the affinity was reduced, and this was attributed to the conformational changes between the single and the multiple peptide repeats [23,32].

### 3.4. Binding Orientation of Truncated Derivative 3 × LPG

**Figure 5:**
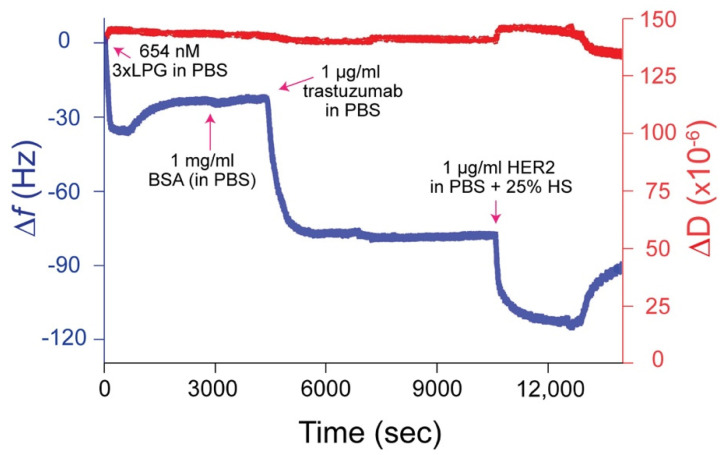
Representative ∆*f* and ∆D versus time plot for 3 × LPG binding to silica followed by the immobilization of trastuzumab and the HER2 antigen spiked in 25% human serum (HS) measured at the 3rd overtone by QCM-D. The measurements were performed at a flow rate of 150 µL/mL at 22 °C. 1 × PBS rinses were applied between protein injections.

In order to assess whether 3 × LPG binds to silica surfaces with its antibody-binding region available to bind an antibody, we used QCM-D to study the binding interaction between silica-bound 3 × LPG and the trastuzumab antibody followed by the ability of the 3 × LPG–trastuzumab complex to detect the HER2 antigen in human serum, a complex biological fluid, as depicted in the ∆*f* and ∆D vs. time sensorgram (Figure 5). The ∆*f* and ∆D values indicated that 3 × LPG bound to the silica, as observed in Figure 4c, and was able to support the binding of 1000 ng/cm^2^ trastuzumab. This immobilized 3×LPG-trastuzumab complex was then able to bind the HER2 antigen that was spiked into 25% human serum. The mass of trastuzumab immobilized to 3 × LPG was approximately 1000 ng/cm^2^ to which approximately 1250 ng/cm^2^ HER2 antigen was immobilized (Table 4). These results indicated less immobilization than reported for 4 × LPG [31]. These results are in accordance with the stoichiometric data presented above, where as many as twice the number of 4 × LPG molecules were bound on the silica surface per cm^2^ as compared to 3 × LPG.

### 3.5. Physiochemical Properties of 4 × LPG Derivatives and Linker Repeats

Supplementary Table S1 (Supplementary Materials) summarizes the physiochemical properties (molecular weight, charge, hydropathicity, and isoelectric point) of the 4 × LPG derivatives and the different linker repeats. The surface of silica is negative and the adsorption of proteins to its surface is influenced by the sum of attraction and repulsion electrostatic forces. The adsorption of proteins will be driven mainly by Coulomb’s electrostatic attractions at pH values below the pI (proteins will carry an overall positive charge). However, absorption will be driven by the sum of the Columbic repulsion and attractive forces (e.g. hydrophobic interactions) at pH values above the pI as proteins and silica will be negatively charged. At pH 7.0 (above pI), PG and all the derivatives (Table S1, Supplementary Materials) carry an overall negative charge. Considering the charge contribution by the linker region alone, the positive charge increases with oligomerization. Accordingly, 4 × L is highly positively charged at pH 7.0 (charge of 12.9) when compared to PG (charge of –14). Silica has a negatively charged surface for the interaction of positively charged molecules, and this may indicate that the linker oligomerization and the concomitant increase in positive charge are the main drive for a strong binding to silica through electrostatic attraction forces. Taniguchi et al. [41] reported that the binding of a silica-binding tag (Si-Tag) was pH-dependent. Due to the presence of 63 positively charged amino acid residues, the peptide displayed a pI of 10.9, and a maximum binding to the silica surface was obtained at pH 8.0, when the Si-tag carried a net positive charge. Sarikaya et al. [42] found that the high affinity of polypeptides to inorganic surfaces was the result of a net high charge and a large proportion of basic amino acid residues.

### 3.6. Introduction of a (GGGGS)_n_ Linker

**Figure 6:**
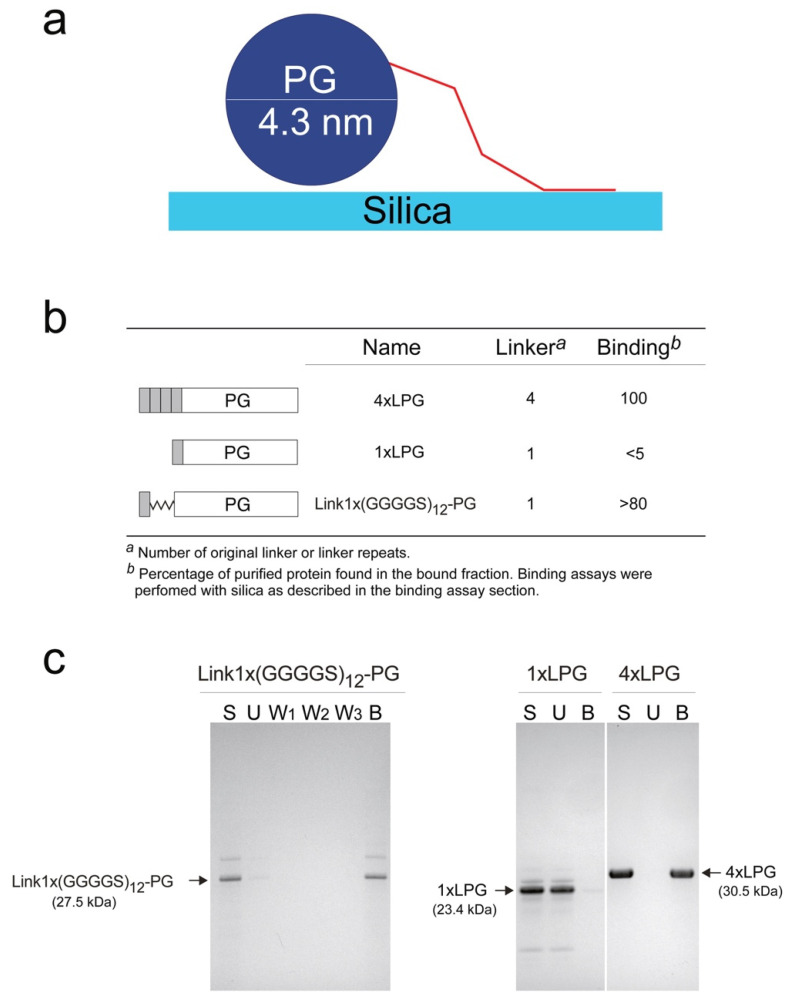
(**a**) Schematic diagram illustrating relative sizes of components assuming ~2 nm per 21 residues of each linker sequence [44] (**b**) Architecture of the fusion protein with and without the glycine-rich spacer and their subsequent zeolite-binding assays. (**c**) sodium dodecyl sulphate (SDS)-polyacrylamide gel electrophoresis (PAGE) of silica binding assays. Arrows indicate position of Link1 × (GGGGS)_12_-PG, 1 × LPG, and 4 × LPG on the gel. 1 × LPG and 4 × LPG SDS-PAGE data in (**b**) and (**c**) were from [1].

Although the linker sequence is important for the binding of 3 × LPG and 4 × LPG to silica, it is not clear whether 4 × LPG binds better simply because it has a repeat of the binding peptide or simply because it is longer. Figure 6 shows a schematic diagram of the relative sizes of the components, which suggests that the sequence of the 3^rd^ repeat unit may be less important than its length. We, therefore, replaced the first three linker units with a glycine-rich flexible soluble sequence (GGGGS)_12_ [43] (Figure 6b) by expressing the fusion protein Link1 × (GGGGS)_12_-PG recombinantly in *E. coli*. The purified Link1 × (GGGGS)_12_-PG has the same pI (4.69), overall charge at pH 7.0 (–13.8), and very similar grand average hydropathicity (–0.64) as 1 × LPG (Table S1, Supplementary Materials). The silica binding assay is a well-established technique [1,6], which allows the semi-quantitative percentage of Link1 × (GGGGS)_12_-PG that remained bound to silica after several wash steps to be determined. Silica binding assays (Figure 6c) indicated that the length of the repeat sequence is an important factor for its solid binding. The insertion of the synthetic (GGGGS)_12_ sequence into 1 × LPG dramatically increases its binding affinity from below the level of quantitation (Figure 4) to >80%. These qualitative initial results seem to indicate an important role for the length of the peptide (distance from the repeats) in an efficient binding to silica. The CD data, which showed that the linkers, as well as some of the N-terminus residues, were unfolded, support the fact that a key role of the middle linkers is simply to let the “fishing hook” of the final silica binding peptide meet the surface. However, in its current form, this experiment shows that only introducing the equivalent length of three linker repeats in the form of (GGGGS)_12_ to 1 × LPG (Link1× (GGGGS)_12_-PG) is enough to restore the binding affinity towards silica almost to the same level as 4 × LPG [1].

The introduction of (GGGGS)_12_ to 1 × LPG results in Link1 × (GGGGS)_12_-PG, which seems to function as an analogue of 4 × LPG but carries only a single sequence of the silica-binding peptide rather than four repeats. This (GGGGS)_12_ spacer addition was enough to convert 1 × LPG, which displayed negligible binding to silica in the silica binding assays [1], into a fusion protein displaying at least qualitatively comparable binding affinity to silica as 4 × LPG [1]. In terms of linker oligomerization, the additional three tandem repeats of the linker may just act as spacer regions that allow the single linker sequence to interact with the silica surface. SBPs are usually present in an unfolded state and adsorption and conformational adaptation to the substrate binding interface are fast and simultaneous events [45]. Over 76% of the amino acid residues of 4 × LPG have the tendency to promote structural disorder [46] and confer low overall hydrophobicity and high net charge, which have been reported for other polypeptide sequences exhibiting affinity to inorganic materials [44]. Thus, the sequence repeats may provide enhanced flexibility and plasticity to adopt an optimal conformation for binding substrates with differently shaped topologies or surfaces.

## 4. Conclusions

There is increased evidence of the contribution of mineral-templated self-assembling systems, including biomolecular self-assemblies of peptides, on the origins of life on Earth and the complexity of early livings systems [47]. Recently, SBPs have been used increasingly as molecular building blocks in several nanobiotechnological applications and have become key molecules for the engineering of biomimetic and bioinspired materials. More biophysical and quantitate analysis of the interaction between SBPs and their inorganic substrates are required to understand their fundamental binding mechanism and expand their range of practical applications. The bifunctional fusion protein 4 × LPG contains a linker made of a 4 × 21-amino acid sequence repeat that displays high binding affinity towards silica. CD and fluorescence spectroscopy studies indicated no apparent negative effect to the secondary structure and chemical stability of the partner PG upon truncation of the associated linker sequence repeats. However, QCM-D analyses suggested that a linker oligomerization is an important parameter for a strong and stable binding to the silica. Removal of the linker sequence below three repeats drastically reduced (2 × LPG) or completely abolished (1 × LPG) the silica-binding property of the 4 × LPG. A 12-repeat glycine rich spacer (GGGGS)_12_ fused between the single linker sequence and PG (1 × LPG) to create a fusion protein of the same length as 4 × LPG with 1×L available for surface binding, Link1×(GGGGS)_12_-PG, restored the silica binding property of the truncated derivative 1 × LPG. The CD data, which showed that the linkers, as well as some of the N-terminus residues, were unfolded, support the conclusion that a key role of the middle linkers is simply to let the “fishing hook” of the final silica binding peptide meet the surface. These preliminary qualitative data provide further motivation to investigate not only oligomerization but also the length of the linker as potentially important parameters for strong silica binding. The results presented here also suggest that SBP oligomerization or incorporation of flexible interdomain linkers like (GGGGS)_n_ can be used to tailor the adsorption and affinity of a given sequence to a solid material. This provides an additional manipulation tool for future applications requiring surface-specific SBPs that act as molecular linkers and assemblers.

## Figures and Tables

**Table 1:** SOMSpec secondary structure estimates for all proteins. The first section is for the data sets for the experimental proteins. The second section is for the proteins where the optimized amount of random coil has been removed. The final section is for the reconstructed proteins.

	α-Helix	β-Sheet	Bends	Turns	Other	NRMSD
PG	0.38	0.16	0.16	0.10	0.20	0.01
1 × LPG	0.29	0.21	0.16	0.10	0.25	0.05
2 × LPG	0.23	0.28	0.13	0.10	0.25	0.06
3 × LPG	0.21	0.31	0.13	0.10	0.25	0.07
4 × LPG	0.17	0.36	0.14	0.10	0.23	0.06
Derandomized proteins						
1 × LPG–0.22RC	0.39	0.15	0.17	0.10	0.20	0.03
2 × LPG–0.31RC	0.36	0.14	0.19	0.09	0.23	0.03
3 × LPG–0.36RC	0.36	0.14	0.19	0.09	0.23	0.03
4 × LPG–0.40	0.34	0.17	0.14	0.12	0.23	0.04
Reconstructed proteins						
1 × LPG	0.38	0.16	0.16	0.10	0.34	0.38
2 × LPG	0.30	0.12	0.13	0.08	0.38	0.30
3 × LPG	0.25	0.10	0.13	0.06	0.47	0.25
4 × LPG	0.23	0.09	0.12	0.06	0.51	0.23

**Table 2:** Numbers of residues in the proteins (notation as in Table 1).

	α-Helix	β-Sheet	Bends	Turns	Other	Total
PG	75	31	31	20	39	196
Derandomized proteins						
1 × LPG–0.22RC	65	25	28	17	33	168
2 × LPG–0.31RC	59	23	30	14	37	163
3 × LPG–0.36RC	59	23	31	14	38	164
4 × LPG–0.40	56	28	24	20	39	167
Reconstructed proteins						
1 × LPG	65	25	28	17	81	215
2 × LPG	59	23	30	14	110	236
3 × LPG	59	23	31	14	130	257
4 × LPG	56	28	24	20	150	278

**Table 3:** Viscoelastic parameters for 3 × LPG–silica binding interactions obtained by fitting the QCM-D data using Voigt model. Data presented as mean ± error (n = 3).

	Thickness (@R_eq_) *^a^* (nm)	Thickness (@k_d_) *^b^* (nm)	Mass Deposited (@R_eq_) (×10^8^ ng/cm^2^)	Mass Deposited (@k_d_) (×10^8^ ng/cm^2^)	Viscosity (@R_eq_) × 10^−4^ (kg/ms)	Viscosity (@R_eq_) × 10^−4^ (kg/ms)
3 × LPG/SiO_2_	5.76 ± 0.15	4.36 ± 0.05	680 ± 34	589 ± 24	19.7 ± 0.4	17.56 ± 0.15

*^a^* @Req, frequency shift at equilibrium; *^b^* @k_d_, saturation achieved after dissociation.

**Table 4:** Viscoelastic parameters for the interaction between silica-bound 3 × LPG and trastuzumab followed by HER2 binding.

Immobilized Truncated Derivative	Thickness of Bound Protein (nm)	Mass of Protein Deposited (ng/cm^2^)	Thickness of Bound Trastuzumab (nm)	Mass Deposited for Bound Trastuzumab (ng/cm^2^)	Thickness of Bound HER2 (nm)	Mass deposited for Bound HER2 (ng/cm^2^)
3 × LPG	5.76 ± 0.22	681 ± 32	15.0 ± 0.20	1019 ± 48	20.0 ± 0.4	1251 ± 51

## Supplementary Materials

The following are available online at https://www.mdpi.com/2079-4991/10/6/1070/s1, Equation S1: Derivation Equation (1) to determine derandomized molar residue Δε of derivatives, Figure S1: SOMSpec output for PG, 1 × LPG, 2 × LPG, 3 × LPG, and 4 × LPG, Figure S2: ΔD vs. time for the 3^rd^ overtone of the absorbed 1 × LPG and 2 × LPG on silica-coated QCM-D crystals at various concentrations (3.27–654 nM), Figure S3: Langmuir adsorption isotherm for the adsorption of 3 × LPG to silica surface, Figure S4: D*f* plot for the binding of 3 × LPG to silica, Table S1: Truncated derivatives of 4 × LPG with different linker repeats and their physicochemical properties.
