# Supplementary material for: The Effect of Oligomerization on A Solid-Binding Peptide Binding to Silica-Based Materials
Source: Nanomaterials (Basel). 2020 May 30;10(6):1070. doi: 10.3390/nano10061070 (PMC7353425; doi:10.3390/nano10061070)
Supplement: Supplementary file 1 [file nanomaterials-10-01070-s001.pdf]

nanomaterials

Supplementary Material

The effect of oligomerization on a solid-binding peptide binding to silica-based materials

Rachit Bansal [1,2], Zehra Elgundi [3], Sophia C. Goodchild [1], Andrew Care [1,2], Megan S. Lord [3], Alison Rodger [1], and Anwar Sunna [1,2,4] *

[1] Department of Molecular Sciences, Macquarie University, Sydney, NSW 2109, Australia ; rachit.bansal@hdr.mq.edu.au (R.B); andrew.care@mq.edu.au (A.C); sophia.goodchild@mq.edu.au (S.C.G); alison.rodger@mq.edu.au (A.R); anwar.sunna@mq.edu.au (A.S)

[2] ARC Centre of Excellence for Nanoscale Biophotonics, Macquarie University, Sydney, NSW 2109, Australia

[3] Graduate School of Biomedical Engineering, University of New South Wales, Sydney, NSW 2052, Australia ; z.elgundi@unsw.edu.au (Z.E) ; m.lord@unsw.edu.au (M.S.L)

[4] Biomolecular Discovery Research Centre, Macquarie University, Sydney, NSW 2109, Australia

* Correspondence: anwar.sunna@mq.edu.au; Tel.: +612-9850-4220

$$\Delta\varepsilon_{\text{de-randomised}} = \frac{\Delta\varepsilon_{\text{exp}} - \frac{\#_{RC}}{\#_{\text{exp}}}\Delta\varepsilon_{RC}}{1-\frac{\#_{RC}}{\#_{\text{exp}}}} \quad (1)$$

$$\Delta\varepsilon_{\text{exp}} = \frac{\#_{\text{de-randomised}}\Delta\varepsilon_{\text{de-randomised}} + \#_{RC}\Delta\varepsilon_{RC}}{\#_{\text{de-randomised}} + \#_{RC}} = \frac{\#_{\text{de-randomised}}\Delta\varepsilon_{\text{derandomised}} + \#_{RC}\Delta\varepsilon_{RC}}{\#_{\text{exp}}}$$

$$\Delta\varepsilon_{\text{exp}}(\#_{\text{de-randomised}} + \#_{RC}) = \#_{\text{de-randomised}}\Delta\varepsilon_{\text{de-randomised}} + \#_{RC}\Delta\varepsilon_{RC}$$

$$\Delta\varepsilon_{\text{de-randomised}} = \frac{\Delta\varepsilon_{\text{exp}}(\#_{\text{de-randomised}} + \#_{RC}) - \#_{RC}\Delta\varepsilon_{RC}}{\#_{\text{de-randomised}}}$$

$$= \frac{\#_{\text{exp}}(\Delta\varepsilon_{\text{exp}} - \frac{\#_{RC}}{\#_{\text{exp}}}\Delta\varepsilon_{RC})}{\#_{\text{exp}} - \#_{RC}}$$

$$= \frac{\Delta\varepsilon_{\text{exp}} - \frac{\#_{RC}}{\#_{\text{exp}}}\Delta\varepsilon_{RC}}{1-\frac{\#_{RC}}{\#_{\text{exp}}}}$$

Equation S1. Derivation of Equation (1) of main text where *RC* denotes random coil, # denotes number of residues, exp denotes the full protein on which data were collected, and de-randomized denotes the core of the protein when the linkers and any extra unfolded residues are removed.

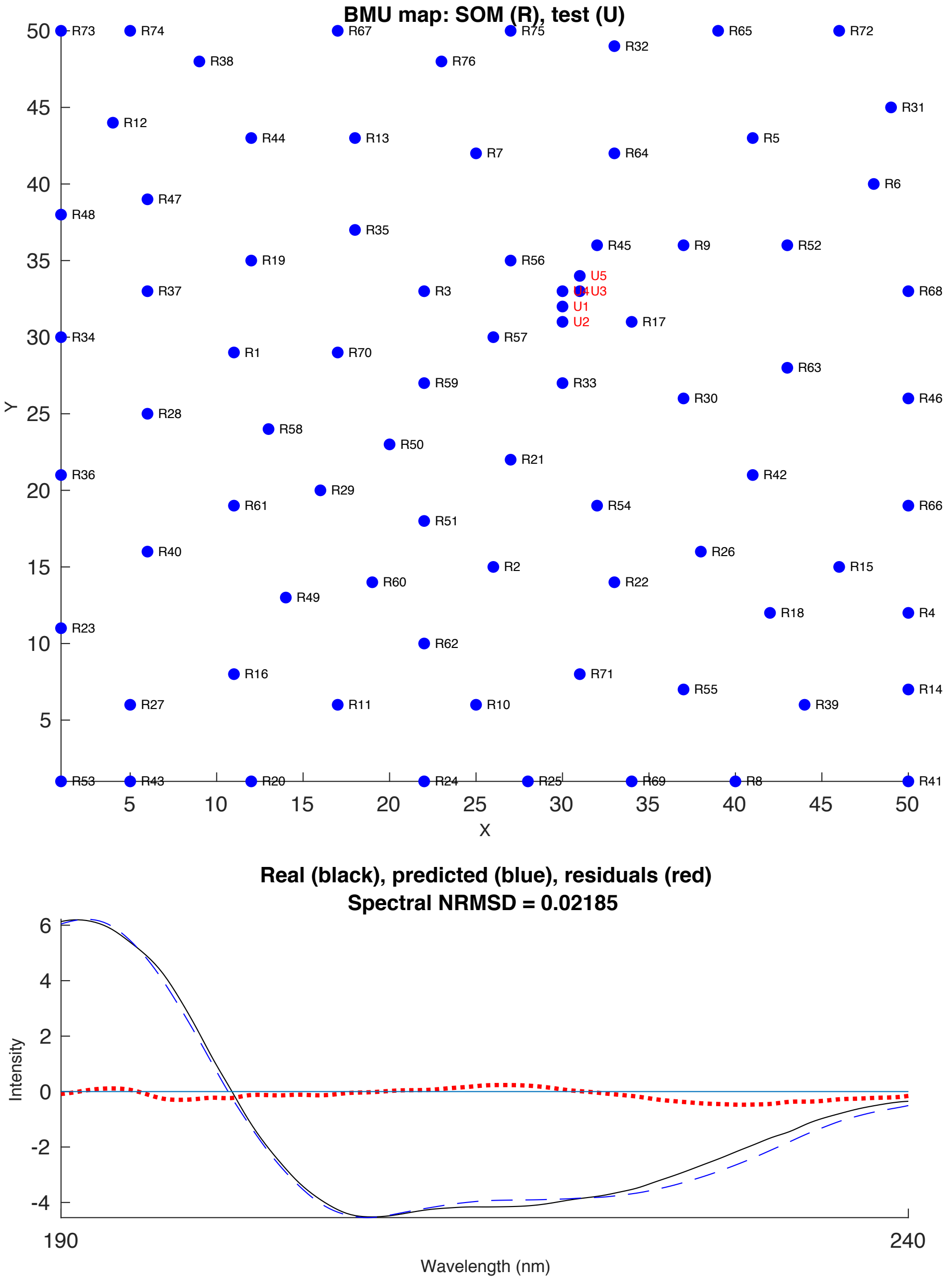
BMU map: SOM (R), test (U)
Y
X
U5
U3
U1
U2
Real (black), predicted (blue), residuals (red)
Spectral NRMSD = 0.02185
Intensity
Wavelength (nm)

(a)

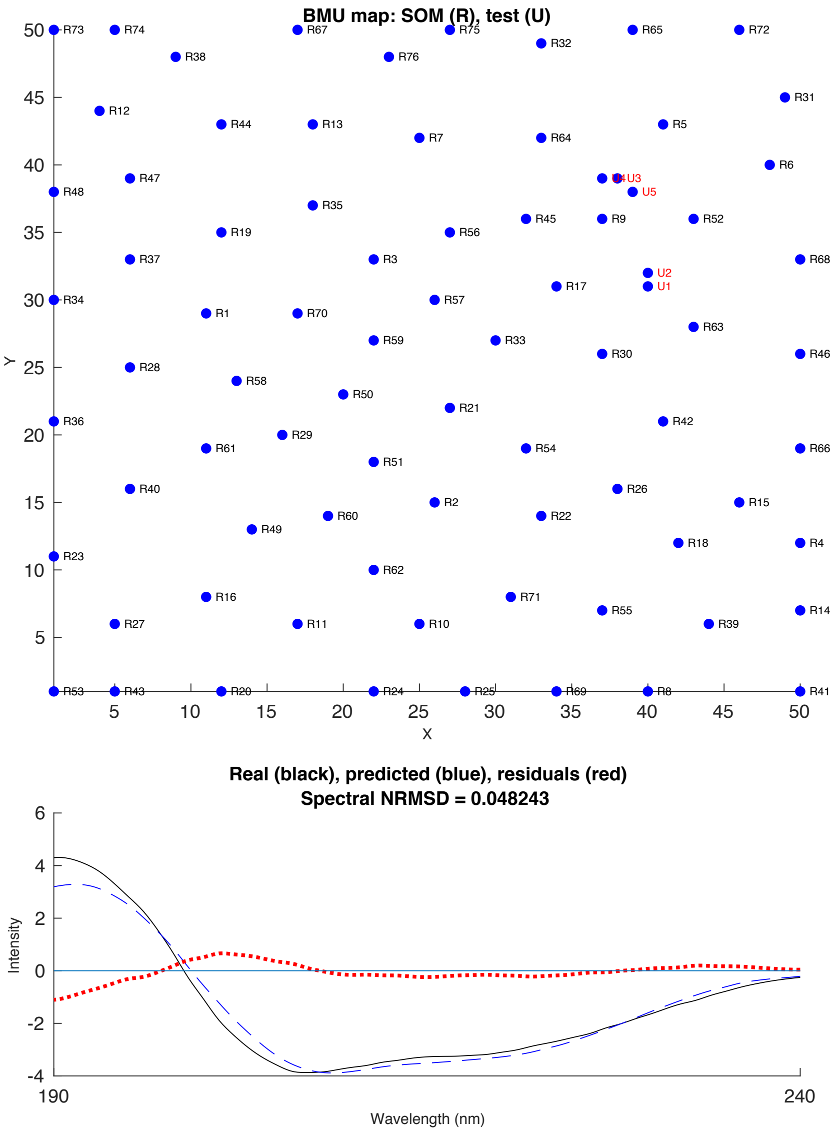
BMU map: SOM (R), test (U)
Y
X
R73
R74
R67
R75
R65
R72
R38
R76
R32
R31
R12
R44
R13
R7
R64
R5
R6
R47
U4U3
U5
R48
R35
R45
R9
R52
R19
R56
R37
R3
R68
U2
U1
R17
R34
R1
R70
R57
R63
R59
R33
R30
R46
R28
R58
R50
R21
R36
R42
R29
R61
R51
R54
R66
R40
R26
R15
R2
R60
R22
R49
R18
R4
R23
R62
R16
R71
R55
R14
R27
R11
R10
R39
R53
R43
R20
R24
R25
R69
R8
R41
Real (black), predicted (blue), residuals (red)
Spectral NRMSD = 0.048243
Intensity
Wavelength (nm)

(b)

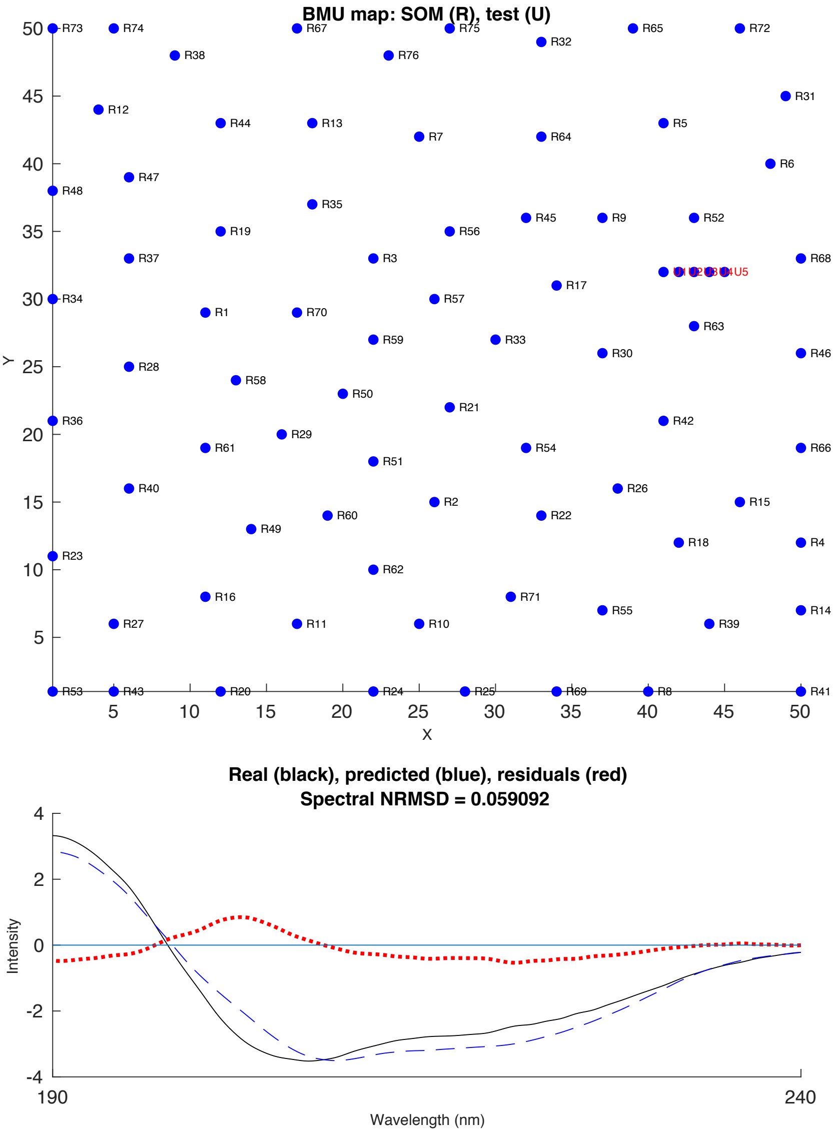
BMU map: SOM (R), test (U)
R73
R74
R67
R75
R65
R72
R38
R76
R32
R31
R12
R44
R13
R7
R64
R5
R6
R47
R48
R35
R45
R9
R52
R19
R56
R37
R3
R68
R17
R34
R57
R1
R70
R63
R59
R33
R30
R46
R28
R58
R50
R21
R36
R42
R29
R61
R51
R54
R66
R40
R26
R2
R15
R60
R22
R49
R18
R4
R23
R62
R16
R71
R55
R14
R27
R11
R10
R39
R53
R43
R20
R24
R25
R69
R8
R41
X
Y
Real (black), predicted (blue), residuals (red)
Spectral NRMSD = 0.059092
Intensity
Wavelength (nm)

(c)

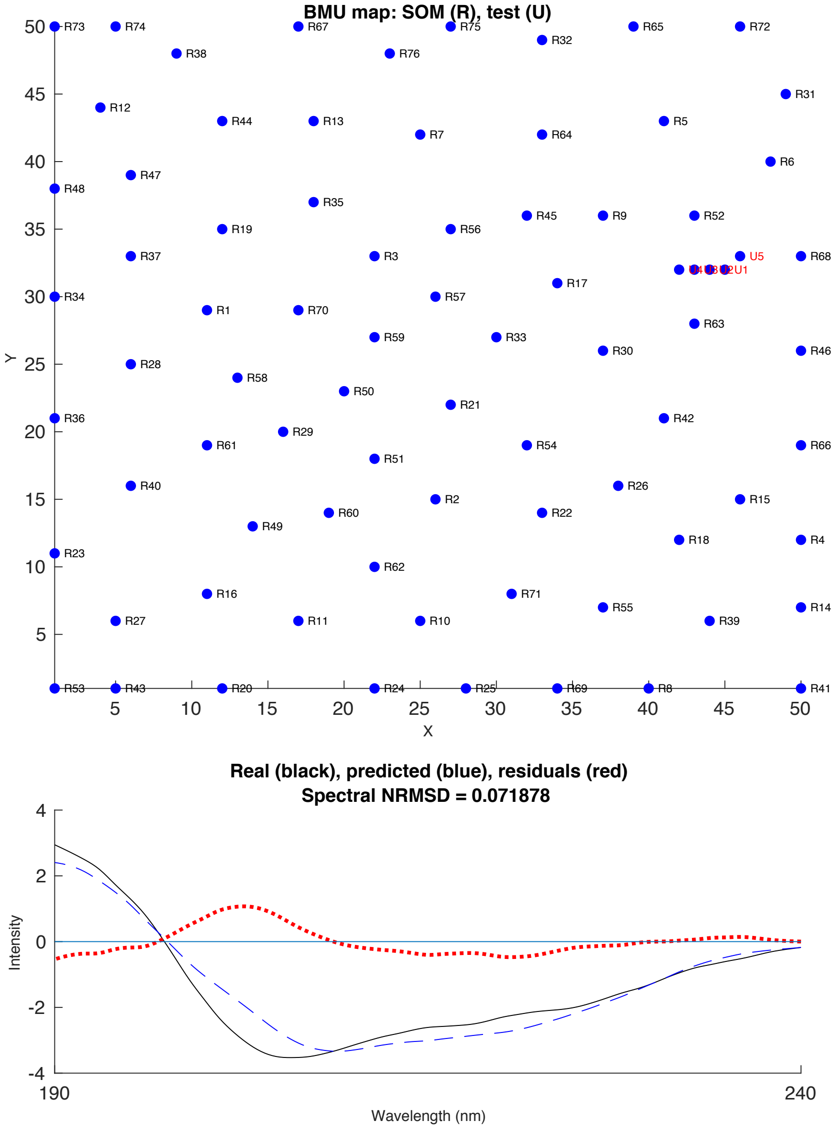
BMU map: SOM (R), test (U)
Y
X
Real (black), predicted (blue), residuals (red)
Spectral NRMSD = 0.071878
Intensity
Wavelength (nm)

(d)

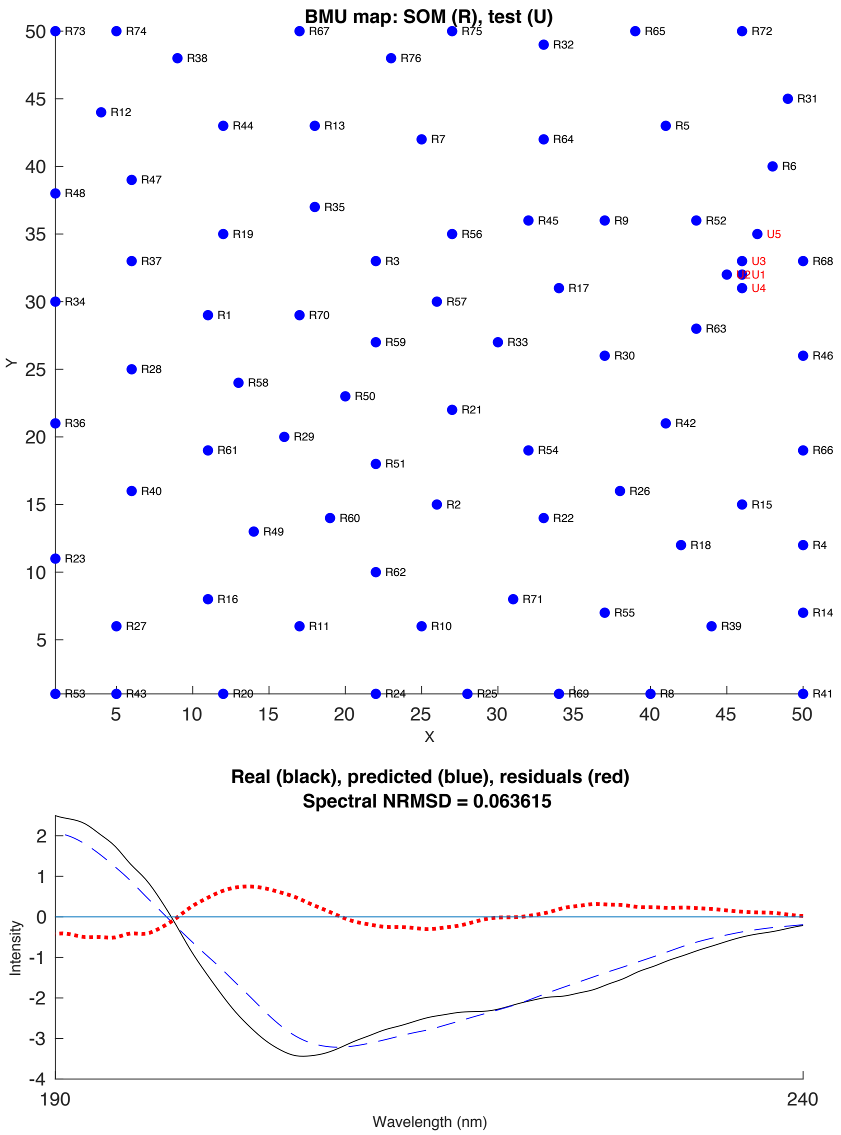
BMU map: SOM (R), test (U)
R73
R74
R38
R67
R75
R76
R32
R65
R72
R31
R12
R44
R13
R7
R64
R5
R6
R47
R48
R35
R45
R9
R52
R19
R56
U5
R37
R3
U3
U1
R68
U4
R17
R34
R57
R1
R70
R63
R59
R33
R30
R46
R28
R58
R50
R21
R36
R42
R29
R61
R54
R66
R51
R40
R26
R2
R15
R60
R22
R49
R18
R4
R23
R62
R16
R71
R55
R14
R27
R11
R10
R39
R53
R43
R20
R24
R25
R69
R8
R41
Y
X
Real (black), predicted (blue), residuals (red)
Spectral NRMSD = 0.063615
Intensity
Wavelength (nm)
190
240

(e)

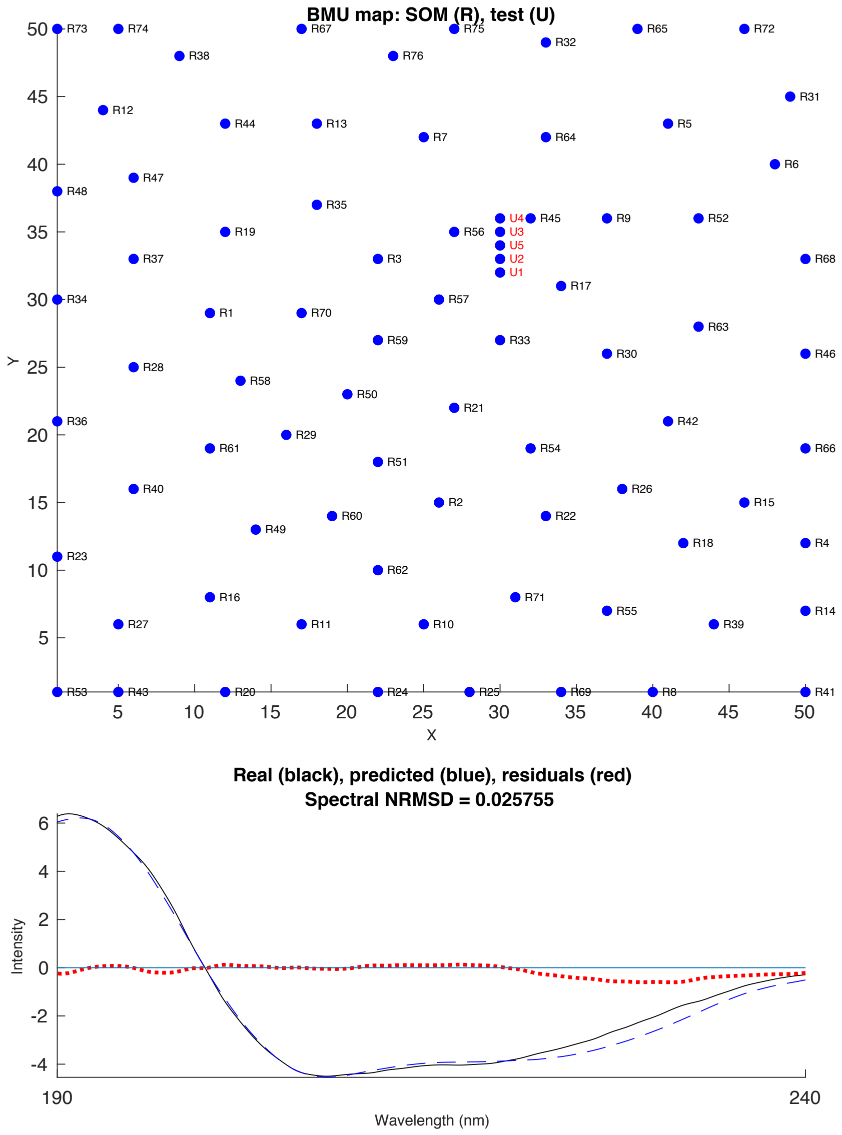
BMU map: SOM (R), test (U)
Y
X
R73 R74 R67 R75 R65 R72
R32
R38 R76
R31
R12
R44 R13 R5
R7 R64
R6
R47
R48
R35
U4 R45 R9 R52
R19 R56 U3
U5
R37 R3 U2 R68
U1
R17
R34 R57
R1 R70
R63
R59 R33
R30 R46
R28
R58
R50
R21
R36 R42
R29
R61 R54 R66
R51
R40 R26
R2 R15
R60 R22
R49
R18 R4
R23
R62
R16 R71
R55 R14
R27 R11 R10 R39
R53 R43 R20 R24 R25 R69 R8 R41
Real (black), predicted (blue), residuals (red)
Spectral NRMSD = 0.025755
Intensity
Wavelength (nm)
190
240

(f)

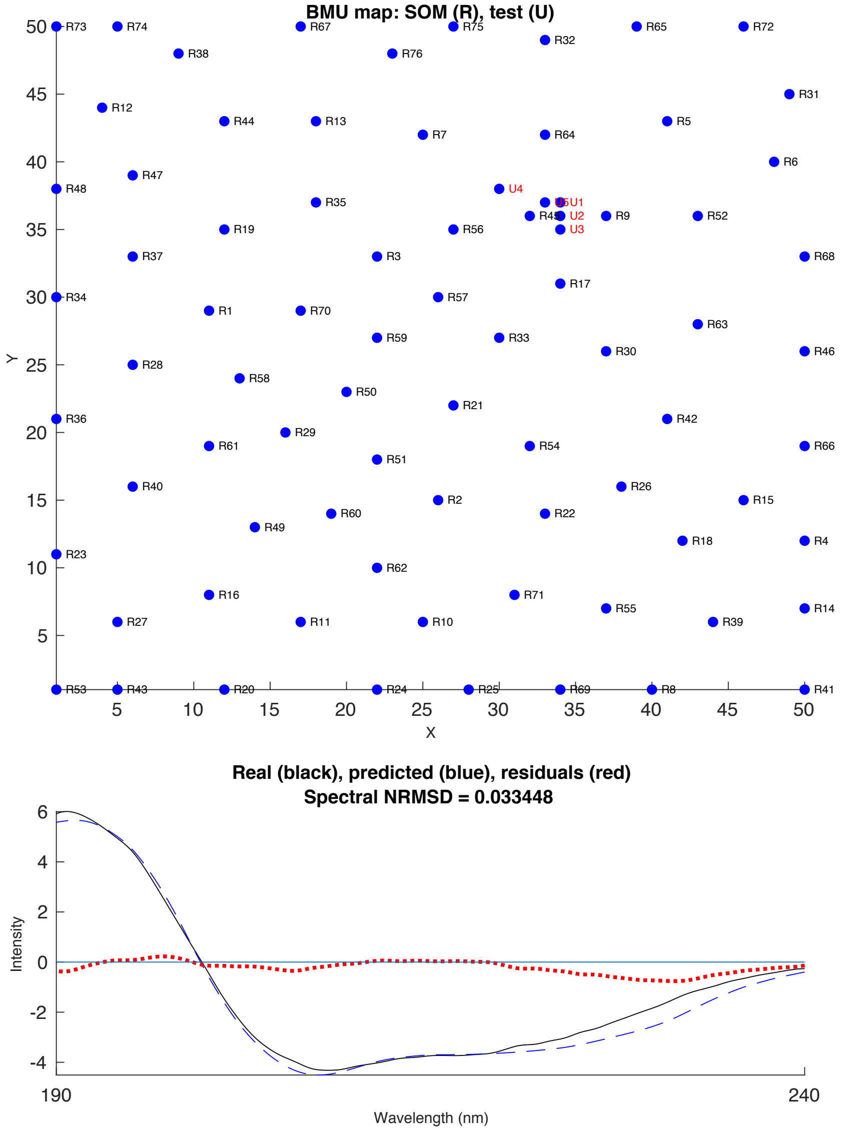
BMU map: SOM (R), test (U)
Y
X
Real (black), predicted (blue), residuals (red)
Spectral NRMSD = 0.033448
Intensity
Wavelength (nm)
190
240

(g)

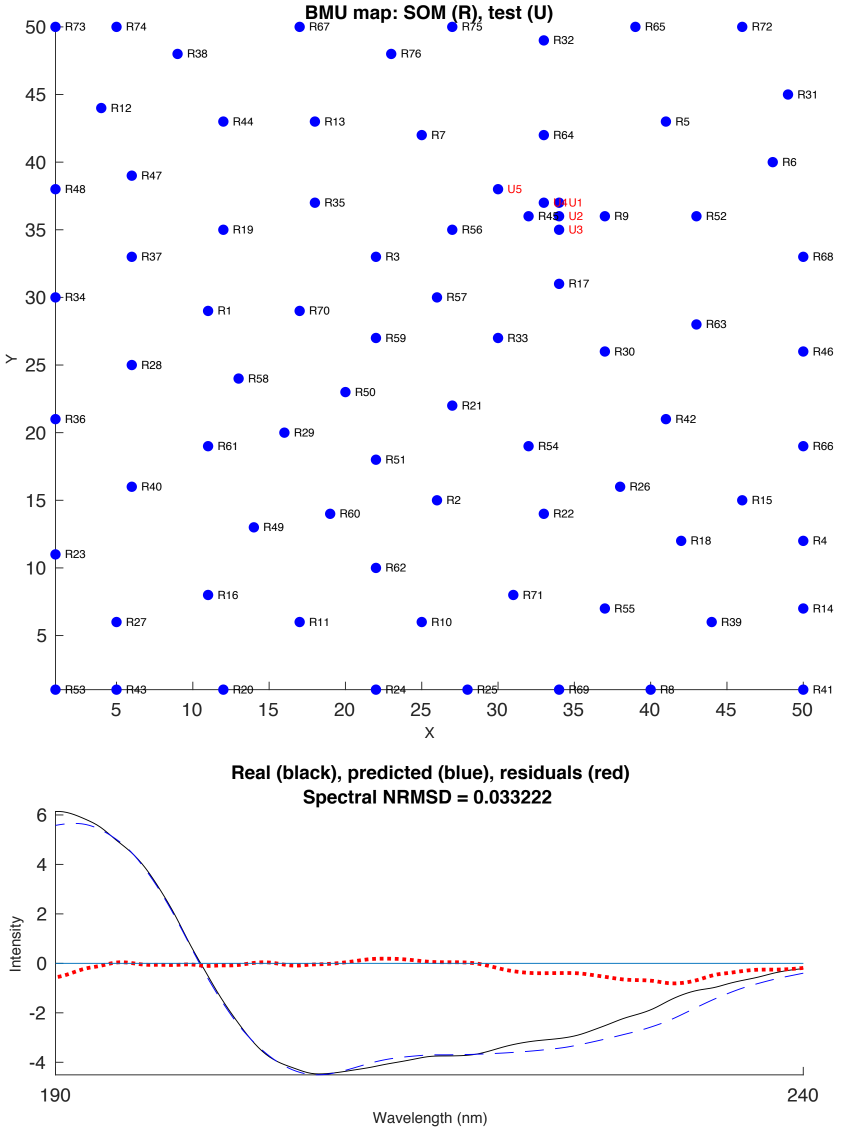
BMU map: SOM (R), test (U)
R73
R74
R67
R75
R65
R72
R32
R38
R76
R31
R12
R44
R13
R5
R7
R64
R6
R47
R48
U5
R35
U1
R45
U2
R9
R52
R19
R56
U3
R37
R3
R68
R17
R34
R57
R1
R70
R63
R59
R33
R30
R46
R28
R58
R50
R21
R36
R42
R29
R61
R54
R66
R51
R40
R26
R2
R15
R60
R22
R49
R18
R4
R23
R62
R16
R71
R55
R14
R27
R11
R10
R39
R53
R43
R20
R24
R25
R69
R8
R41
Y
X
Real (black), predicted (blue), residuals (red)
Spectral NRMSD = 0.033222
Intensity
Wavelength (nm)

(h)

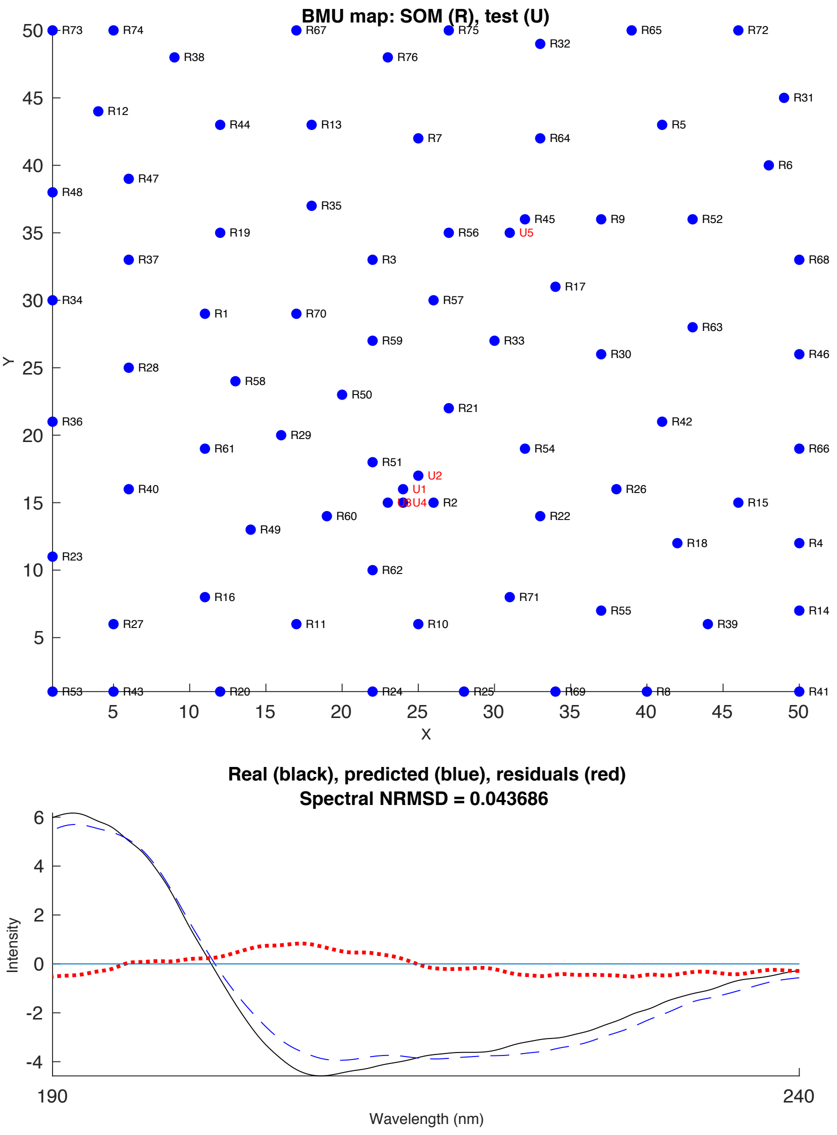

(i)

Figure S1. SOMSpec output for (a) PG, (b) 1×LPG, (c) 2×LPG, (d) 3×LPG, (e) 4×LPG, (f) 1×LPG–0.22 random coil, (g) 2×LPG–0.31 random coil, (h) 3×LPG–0.36 random coil, (i) 4×LPG-0.40 random coil

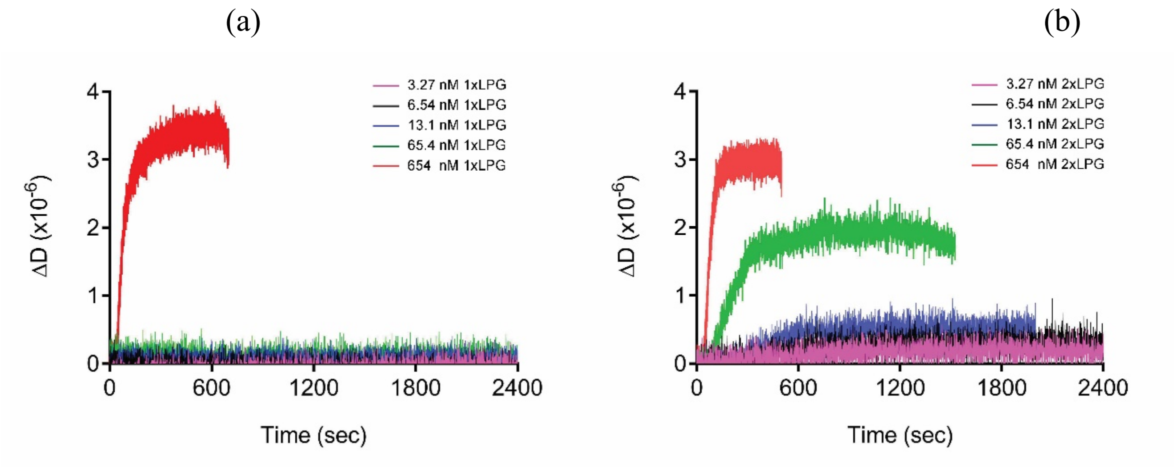

Figure S2. ΔD vs time for the 3rd overtone of the absorbed **(a)** 1xLPG and **(b)** 2xLPG on silica coated QCM-D crystals at various concentrations (3.27–654 nM). The measurement was performed in three steps at a flow rate of 150 µL/mL: baseline formation (1 × PBS buffer), adsorption of protein samples until saturation, and washing (1 × PBS buffer) to remove of unbound protein. Three independent measurements were performed for each concentration.

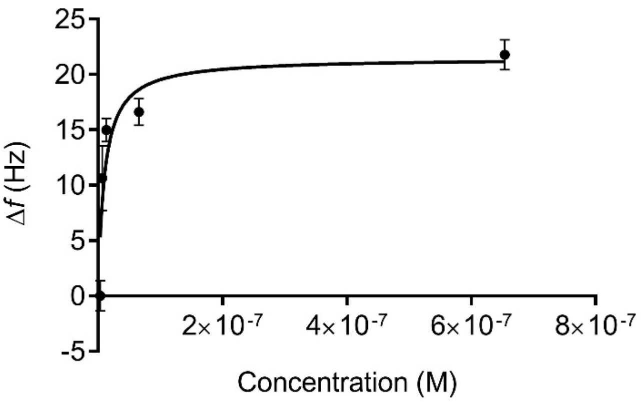

Figure S3. Langmuir adsorption isotherm for the adsorption of 3×LPG to silica surface. The data were fitted as per a single-site specific binding model, giving a binding affinity/equilibrium dissociation constant (K_D)= 53.23±4.5 nM.

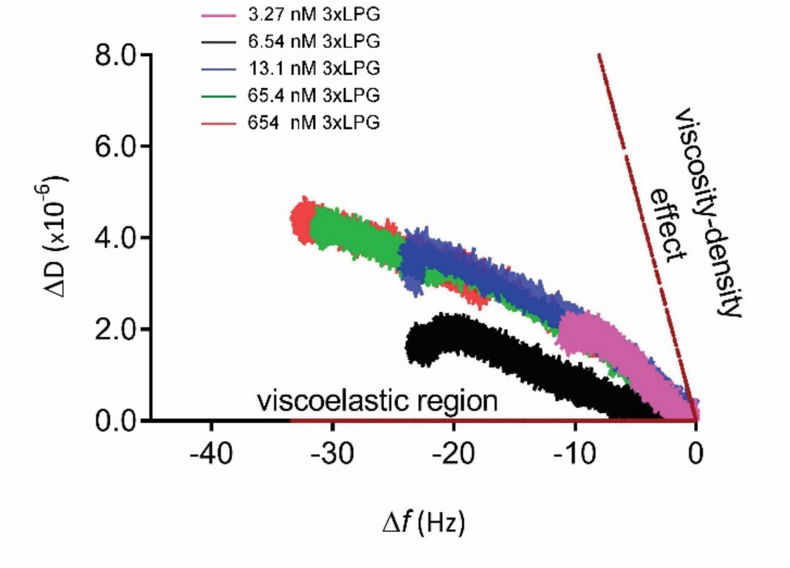

Figure S4. D*f* plot for the binding of 3×LPG to silica . The dotted lines indicate the pure elastic mass and viscosity-density responses indicating the data lie within the viscoelastic region.

Supplementary Table S1. Truncated derivatives of 4×LPG with different linker repeats and their physicochemical properties.

	**Mw (kDa)**[a]	**pI**[b]	**Charge**[c]	**GRAVY**[d]
Protein				
PG	21.363	4.58	–14.0	–0.56
1×LPG	23.443	4.69	–13.8	–0.69
2×LPG	25.797	4.94	–10.5	–0.78
3×LPG	28.151	5.25	–7.3	–0.85
4×LPG	30.504	5.80	-4.1	–0.91
Linker region without PG				
1×L	3.232	10.94	3.2	–1.27
2×L	5.586	11.38	6.4	–1.43
3×L	7.939	11.50	9.6	–1.50
4×L	10.293	11.57	12.9	–1.54

[a] Theoretical molecular weight (Mw)*
[b] Theoretical isoelectric point (pI)*
[c] Theoretical charge at pH 7.0*
[d] Theoretical grand average hydropathicity (GRAVY)†
* Values calculated using Protein calculator v3.4 (http://protcalc.sourceforge.net/)
† Values calculated using Protein GRAVY (https://www.bioinformatics.org/sms2/protein_gravy.html)
